# The Maristán stigma scale: a standardized international measure of the stigma of schizophrenia and other psychoses

**DOI:** 10.1186/1471-244X-14-182

**Published:** 2014-06-18

**Authors:** Sandra Saldivia, Ariadne Runte-Geidel, Pamela Grandón, Francisco Torres-González, Miguel Xavier, Claudio Antonioli, Dinarte A Ballester, Roberto Melipillán, Emiliano Galende, Benjamín Vicente, José Miguel Caldas, Helen Killaspy, Rachel Gibbons, Michael King

**Affiliations:** 1 Department of Psychiatry and Mental Health, Faculty of Medicine, University of Concepcion, Concepcion, Chile; 2 Department of Pedagogy, University of Jaen, Jaen, Spain; 3 Department of Psychology, Faculty of Social Sciences, University of Concepcion, Concepcion, Chile; 4 Centre of Bio-Medical Research in Network of Mental Health (CIBERSAM), Section of Psychiatry and Medical Psychology, University of Granada, Granada, Spain; 5 Faculty of Medical Sciences, University Nova of Lisbon, Lisbon, Portugal; 6 Department of Community Health, University National of Lanus, Buenos Aires, Argentina; 7 University State of Londrina, Londrina, Brazil; 8 Division of Psychiatry, Faculty of Brain Sciences, UCL Medical School, London, UK; 9 Camden and Islington NHS Foundation Trust, London, UK

**Keywords:** Stigma, Questionnaire, Psychometrics, Rating scale schizophrenia

## Abstract

**Background:**

People with schizophrenia face prejudice and discrimination from a number of sources including professionals and families. The degree of stigma perceived and experienced varies across cultures and communities. We aimed to develop a cross-cultural measure of the stigma perceived by people with schizophrenia.

**Method:**

Items for the scale were developed from qualitative group interviews with people with schizophrenia in six countries. The scale was then applied in face-to-face interviews with 164 participants, 103 of which were repeated after 30 days. Principal Axis Factoring and Promax rotation evaluated the structure of the scale; Horn’s parallel combined with bootstrapping determined the number of factors; and intra-class correlation assessed test-retest reliability.

**Results:**

The final scale has 31 items and four factors: informal social networks, socio-institutional, health professionals and self-stigma. Cronbach’s alpha was 0.84 for the Factor 1; 0.81 for Factor 2; 0.74 for Factor 3, and 0.75 for Factor 4. Correlation matrix among factors revealed that most were in the moderate range [0.31-0.49], with the strongest occurring between perception of stigma in the informal network and self-stigma and there was also a weaker correlation between stigma from health professionals and self-stigma. Test-retest reliability was highest for informal networks [ICC 0.76 [0.67 -0.83]] and self-stigma [ICC 0.74 [0.64-0.81]]. There were no significant differences in the scoring due to sex or age. Service users in Argentina had the highest scores in almost all dimensions.

**Conclusions:**

The MARISTAN stigma scale is a reliable measure of the stigma of schizophrenia and related psychoses across several cultures. A confirmatory factor analysis is needed to assess the stability of its factor structure.

## Background

People with schizophrenia and other psychoses face a range of problems, some arising directly from the illness and others from the stigma of the disorder. Stigma can best be understood as the loss of status by, or discrimination of, a person because of an attribute that others evaluate disapprovingly [1]. Stigma complicates recovery, thereby reducing self-esteem and access to social networks [2,3]. It has a particularly severe impact on patients’ quality of life [4]. For this reason stigma has generated considerable interest in recent decades [3,5].

Given that it is the result of a social interaction, stigma’s manifestations vary from one culture to another [6,7]; each society determines what is considered abnormal, how illness is defined and how and where help is sought [8].

An increasing number of studies have assessed stigma and its consequences. Some of them have focused on public attitudes to people with mental illness [9-11], others have considered the perception of the families or carers and professional groups, and others have measured the personal experience of those who suffer stigma [12-14]. Even psychiatrists have been shown to stigmatise patients with mental illness. For example, 42% of psychiatrists surveyed recently in Brazil negatively stereotyped people with schizophrenia, agreed with some restrictions on their civil liberties (e.g. the right to vote) and scored highly on attitudes reflecting prejudice and a need to keep a social distance [15]. Although qualitative studies of interviews with people with mental illness have shown that stigma can occur in a variety of forms [16], the main distinction made by service users is between acts of discrimination and feelings of stigma. The first concerns actual social rejection, while the second refers to feelings of inferiority and shame, and fear of provoking negative responses in others [16].

A further element is self-stigma when the person seems to accept a negative stereotype and either becomes ashamed or simply hides some element of their make-up that they perceive is unacceptable to others. This happens when the mentally ill person internalizes the stereotypes and negative social attitudes associated with people with mental disorders to the extent that they shape his/her own identity [17,18]. High self-stigma appears to be at the opposite pole to a sense of personal empowerment and control [19]. Self-stigma also occurs in family members, particularly those with full insight into the mental illness and possibly its gravity [20].

Most instruments used in measuring personal stigma of mental illness have considered different ways in which stigma is experienced; some are focused on stigmatisation of the self [3,21], others are based in the direct experience of discrimination [13,22], some on the perception of stigma [23,24], and more recent studies have considered the anticipated discrimination [5,25]. Most instruments evaluate both perception and experience of the stigma and some consider self-stigma [26]. People’s experience of stigma is considered mainly in terms of perceived discrimination and stereotypes about the population with mental illness [26].

Only a few instruments have based the development of their scale on qualitative data about people’s experiences and views. Angermeyer et al. [27] developed a scale from results of focus groups conducted in Germany [27,28]. An instrument developed by Stuart et al. [12] also started with a qualitative approach; it has two scales, one of which assesses the experience of stigma in a number of life’s domains and the other evaluates its impact [12]. Differences in the response format and number of items make it difficult to make comparisons with other instruments and reduce the possibility of exploring the complexity of the experience. Finally, Thornicroft et al. [5] developed an instrument starting with a qualitative step but this step was limited to obtaining the views of people to a number of items already extracted from the literature. The resulting scale, the DISC is a long and complex instrument that measures a number of domains. Its main advantage is that it was developed across a number of European countries [5]. Its disadvantage is lack of detailed psychometric data. A recent systematic review compared instruments developed up until 2009. It is clear in this review that nearly all were developed in a single culture [26].

Few studies have been multi-cultural [5,25]. Some researchers have found that there are no differences on the stigma in different places [29], whereas others show a lower discrimination in certain sites [30]. Cheon and Chiao [31] reaffirm the importance of taking account of cross-cultural factors in the study of stigma, because cultural variations may be important even in societies with low overall stigma [31,32]. Furthermore, given that few cross-cultural studies use the perceptions of affected persons [33], there is the need to focus more on this approach.

The Maristán Network is an international group of mental health professionals who collaborate on research and teaching in Europe and Latin America (http://www.redmaristan.org/). Our objective was to develop an instrument to evaluate stigma in people with schizophrenia and other psychoses that would have validity across cultures. We aimed to construct a broad measure of stigma whose component parts could assess perceived stigma from family, friends, colleagues and professionals, stigma in its more institutional form and self-stigma if it were evident. We did not have the resources to recruit enough people to compare the degree and type of stigma between countries (with appropriate statistical power). Rather, our aim was to include all countries to ensure that the psychometric properties of the instrument would have relevance to all.

## Methods

The method of the overall Maristán study, of which this is a part, is presented in detail elsewhere [34]. In brief, the process began with the collection of data from 46 focus groups in six countries in which 303 respondents participated, including patients with ICD-10 diagnoses of schizophrenia and other psychotic disorders, informal and formal carers. All participants in each focus group were asked to discuss informal care, needs for care and the stigma experienced by patients with schizophrenia. The focus groups were undertaken in 2002 in Argentina, Brazil, Chile, Spain, the United Kingdom and Venezuela. A tree of categories was constructed from the qualitative data for stigma and this tree was the basis on which the items were derived [35]. The questionnaire was first developed in Spanish and a member of each local team in Lisbon and London translated the Spanish version after which both the original and the translation were reviewed by the research team. Three expert groups (consisting of 23 people in all), each communicating in one of three languages [Spanish, Portuguese or English], then agreed a first version of the instrument. This penultimate version was then sent back to the expert group members who carried out a final assessment by scoring the relevance of each statement from 1 (not at all relevant) up to 5 (highly relevant); the score three, as the median, was established as the cut-off point and the items that scored less were considered of little or no relevance [34]. This led to a final draft of the questionnaire, which was tested in a new sample of patients in Buenos Aires, Argentina; Porto Alegre, Brazil; Concepción, Chile; Granada, Spain; Lisbon, Portugal; and London, England, using the appropriate versions in Spanish, Portuguese and English.

### Participants

The aim in this final phase of the study was to recruit up to 30 patients from each country, aged 18 years and over whose diagnoses of schizophrenia and related psychoses [F20-F29, International Classification of Disease version 10] was confirmed by the responsible psychiatrists. Participants were attending outpatient psychiatric services or day centres, had experienced a minimum of three years since the first known contact with services and had at least one year of continuous contact with the service. We chose three years to be sure that the diagnosis was established and stable, and for sufficient time to have passed for patients to have lived with the diagnosis and experienced stigma. Participants with moderate or profound intellectual disability and those with a history of illness of over 15 years since first contact with services were excluded. The latter was to restrict the sample to people with lesser degrees of institutionalisation, who receive care in model of community mental health. It was also our clinical observation that patients with longer term illness become accustomed or resigned to stigma and may be less likely to notice its effects on a day-to-day basis. Participants were identified from the clinical service registers in each participating centre. In the UK a key clinician invited patients to participate and if they agreed they were put in contact with a researcher who confirmed the diagnosis and inclusion criteria. In other countries researchers used a mixture of direct contact with patients and recruitment via clinicians. A written informed consent was obtained in keeping with each country’s guidance for good clinical practice in research. The study was approved by appropriate research ethical committees of the Faculties of Medicine and local health services in each country (Ethic Committees in National University of Lanus, Argentina, Maternal and Children’s Hospital Presidente Vargas, Porto Alegre, Brazil, Faculty of Medical Sciences in Nova University of Lisbon, Portugal, Faculty of Medicine in University of Concepcion, Chile, Ethic Committee in Human Research (CEIH) in Granada, Spain; and Camden and Islington Local Research Ethics Committee in UCL Medical School, UK).

### Instrument

The first draft of the Maristán Questionnaire that was developed for testing consisted of 38 statements to which the respondents indicate their degree of agreement. The statements concerned stigma in personal, family and social life, the attitudes of health professionals, and stigma in the public sphere and work. The format was a Likert scale with a score of 1 indicating complete disagreement and 7 indicating complete agreement with each statement. Where appropriate, the direction of scoring was reversed so that a high score indicates a high level of stigma. In all countries, the questionnaire was read out by an interviewer to make sure the meaning was clear.

### Procedure

As described, participants took part in individual face-to-face interviews in which the questionnaire was delivered by professionals who had clinical or other experience in the care of people with severe mental illness. All of the interviewees were invited to answer the questionnaire again after 30 days in order to estimate temporal stability. Each investigator kept field notes regarding the application of the questionnaire, including the degree of comprehension of each statement, the language terms used, views on the length of the interviews and any adverse comments about them.

### Data analysis

The analysis was undertaken by means of SPSS, version 11.0. An exploratory factor analysis was used to evaluate the structure of the scale, using Principal Axis Factoring [PAF], followed by a Promax rotation. This approach is one of the most recommended strategies for analysis of the structure of a rating scale [36-38]. In order to determine the number of factors to extract we used Horn’s parallel analysis combined with sample selection or bootstrapping [39-41]. This analysis addresses weaknesses in more traditional factor analysis. For example, the Kaiser-Guttman rule dictates that all factors with an eigenvalue of more than 1.0 should be retained but ignores the possibility of random variation in eigenvalues obtained from any one sample population. Similarly, in the Catell analysis of the scree plot to determine the number of factors to be retained, it is often not possible to make an unequivocal decision about the cut-point, which leads to indecision on how many factors to retain [39,42-45]. Pearson’s product moment correlations were used to obtain correlations between factors. Cronbach’s alpha was used to estimate internal consistency and the intra-class correlation coefficient (with 95% confidence intervals) for test-retest reliability. A series of ANOVA were used to evaluate differences in factor scores between groups of patients.

## Results

164 interviews were undertaken, of which 103 repeated the scale after about 30 days. Socio-demographic characteristics of participants in each country are given in Table 1. 60.4% of participants were men, 82.2% were single, 54.6% had completed secondary schooling and 54.9% were in receipt of Social Security payments. Mean age was 39 years (sd 9.8), while mean years of illness was 12.8 (sd 8.8). Patients interviewed in Argentina were older, had greater involvement in work (27.8%) and included fewer single persons (61.1%). Brazil was the only country where the proportions of women and patients with only basic education were the greatest, 72.4% and 55.2% respectively. Patients in the sample from Spain had a low level of education (45% with basic schooling), and Argentina and Portugal had the lowest proportion of patients classified as disabled by Social Security.

**Table 1:** **Sample socio**-**demographic characteristics by country**

**Characteristic**	**Argentina**	**Brazil**	**Chile**	**Portugal**	**Spain**	**U.K**.	**Total**
	18 (11.0%)	30 (18.3%)	30 (18.3%)	20 (12.2%)	20 (12.2%)	46 (28.0%)	164 (100.0%)
Male	9 (50.0%)	8 (27.6%)	25 (83.3%)	11 (55.0%)	14 (70.0%)	32 (69.6%)	99 (60.7%)
Age mean (sd)	45.7 (6.4)	36.7 (9.2)	39.6 (10.1)	34.4 (8.3)	38.5 (8.4)	41.2 (10.9)	39.41 (9.8)
Single	11 (61.1%)	25 (86.2%)	25 (83.3%)	16 (80.0%)	17 (85.0%)	40 (87.0%)	134 (82.2%)
Education							
None	5 (27.8%)						5 (3.1%)
Elementary	2 (11.1%)	16 (55.2%)	9 (30.0%)	2 (10.0%)	9 (45.0%)	3 (6.5%)	41 (25.2%)
Secondary	10 (55.6%)	11 (37.9%)	17 (56.7%)	14 (70.0%)	7 (35.0%)	30 (65.2%)	89 (54.6%)
Higher	1 (5.6%)	2 (6.9%)	4 (13.3%)	4 (20.0%)	4 (20.0%)	13 (28.3%)	28 (17.2%)
Employment							
In work	5 (27.8%)	1 (3.8%)	3 (10.0%)	3 (15.0%)	1 (5.0%)	3 (6.5%)	16 (10.0%)
Unemployed	7 (38.9%)	9 (34.6%)	3 (10.0%)	11 (55.0%)	6 (30.0%)	3 (6.5%)	39 (24.4%)
Social security benefits	6 (33.3%)	16 (61.5%)	24 (80.0%)	6 (30.0%)	13 (65.0%)	40 (87.0%)	105 (65.0%)
Years of illness	18.2 (±7.2)	15.8 (±7.9)	19.3 (±11.3)	9.3 (±4.1)	18.7 (±10.9)	14.1 (±10.5)	12.8 (±8.8)

Results obtained from Horn’s Parallel Analysis on 5,000 bootstrap samples revealed that only the first four eigenvalues of the correlation matrix of the stigma scale [eigenvalues 7.86, 2.97, 2.07, and 1.85] were greater than the 95 percentile of the eigenvalues obtained from the bootstrap samples [values 2.20, 2.03, 1.91 and 1.81]. This result revealed that there were four main factors in the scale. For the purposes of comparison, application of the Kaiser Guttman criterion identified 12 factors, while the analysis utilizing the scree plot revealed a sharp break after the second factor.

Given the above, it was decided to extract four factors followed by a Promax rotation. The analysis of the pattern matrix revealed that six items had coefficients less than 0.3 on any of the four factors and thus these were removed and the extraction repeated. The results of the second analysis showed that one additional item had a coefficient of less than 0.3. This item was also removed and the analysis repeated once again, resulting in all remaining items having coefficients greater than 0.3. Thus this was accepted as the final factor solution for interpretation [see Table 2].

**Table 2:** Pattern matrix for the MARISTAN Stigma Scale

**Item**		**Informal networks**	**Socio**-**institutional**	**Health professionals**	**Self-stigma**
10	I have been hidden away by my family	***0,*** ***76***	−0,10	0,01	−0,02
16	Due to my illness people close to me have become more distant	***0,*** ***60***	0,02	0,03	−0,08
8	My family doesn’t take me seriously	***0,*** ***59***	0,14	0,06	−0,03
15	People look at me as if I am odd	***0,*** ***57***	0,08	−0,06	0,18
18	People always expect me to behave and talk in an odd way	***0,*** ***57***	−0,04	−0,06	−0,01
12	My family is afraid of me	***0,*** ***56***	−0,20	−0,07	0,02
19	People don’t respect me	***0,*** ***56***	0,04	0,09	0,00
9	I have been ill-treated by my family (abandoned, locked in my own home, etc.).	***0,*** ***48***	−0,15	0,13	0,11
7	My family criticises me for everything	***0,*** ***45***	0,11	0,07	0,04
20	People are not tolerant with me	***0,*** ***44***	0,11	0,16	0,08
11	My family hides my illness from others	***0,*** ***40***	−0,02	0,00	0,20
29	Health Problems of people with mental health problems are not taken seriously, as they tend to be understood as part of the mental illness	0,12	***0,*** ***66***	−0,17	−0,14
28	The opinion of people with mental health problems is not so well considered whenever decisions about their treatment (hospital admission) are taken, unlike in the case of other illnesses	0,05	***0,*** ***62***	−0,02	−0,13
35	There are not enough services for people with mental health problems	−0,09	***0,*** ***58***	0,12	−0,05
31	Mental health services have fewer staff than other health services	−0,30	***0,*** ***57***	0,13	0,18
26	Medical services try to avoid having to deal with people with mental health problems	0,10	***0,*** ***55***	0,05	−0,02
27	The emergency services don’t look after people with mental health problems in the same way as people with other illnesses.	0,09	***0,*** ***53***	−0,01	0,04
33	Government policy has not supported the development of mental health services compared to other health services	−0,12	***0,*** ***46***	0,06	0,13
14	People use unpleasant words when talking about people with mental health problems	0,19	***0,*** ***43***	−0,14	0,06
34	Mental health law excessively restricts the rights of people with mental health problems	−0,11	***0,*** ***42***	−0,08	0,23
30	Mental health facilities are in a worse state than other health service facilities.	−0,12	***0,*** ***41***	0,22	0,14
21	The media portray a poor image of people with mental health problems	0,36	***0,*** ***40***	−0,09	−0,11
32	Psychiatric medication is not as well funded as medication for other illnesses	−0,14	***0,*** ***36***	0,25	−0,08
23	Mental health professionals are cold and impersonal towards me	0,11	−0,10	***0,*** ***84***	−0,01
22	General practice staff are cold and impersonal towards me	−0,02	−0,02	***0,*** ***76***	−0,02
24	Professionals from the emergency services are cold and impersonal towards me	0,10	0,20	***0,*** ***53***	−0,06
25	Professionals in psychiatric units are cold and impersonal towards me	0,08	0,27	***0,*** ***33***	−0,04
2	The illness makes me feel inferior	0,09	−0,20	0,11	***0,*** ***76***
1	I avoid relating to others because I feel different	0,03	0,02	−0,05	***0,*** ***67***
3	I’m afraid that I might disappoint others	0,05	0,13	−0,11	***0,*** ***61***
5	At times I think that I am not normal	0,11	0,22	−0,08	***0,*** ***42***

***Italic text***, *it shows items belonging each factor.*

Following this analysis the scale contained 31 items and four factors. We called factor 1 "informal networks” as it contains 11 items on perception of stigmatizing attitudes of people closest to the patient, including the family, partner and friends. The second factor, namely “socio-institutional”, consists of 12 items on the stigma in health services, the communication media, people who have close contact with patients, and mental health law. The third factor “health professionals” was composed of four items concerning cold and impersonal attitudes of professionals with regard to patients. The fourth factor which we called “self-stigma” concerned four items on patients’ feelings of inferiority and low expectations of their own performance.

With regard to internal consistency of each factor, Factor 1 had a Cronbach’s Alpha coefficient of 0.84, with values of corrected item - total correlation ranging from 0.43 to 0.64. Items in the Factor 2 had a Cronbach’s Alpha of 0.81 with corrected item - total correlations from 0.34 to 0.54. Cronbach’s alpha for the Factor 3 was 0.74 with corrected item - total correlations varying between 0.34 and 0.65. Finally, the internal consistency of Factor 4 was 0.75 with corrected item - total correlations ranging from 0.44 to 0.62.

Analyzing the correlation matrix between factors [see Table 3] revealed that most were in the moderate range [0.31-0.49], with the strongest occurring between perception of stigma in the informal network [factor 1] and self-stigma [factor 4]. Conversely there was a weaker correlation between stigma from health professionals [factor 3] and self-stigma [factor 4].

**Table 3:** Pearson correlation between the MARISTAN Stigma Scale total score and factor scores

	**Informal networks**	**Socio**-**institutional**	**Health professionals**	**Self**-**stigma**
Informal networks	1.00	0.42***	0.31***	0.49***
Socio-institutional		1.00	0.41***	0.32***
Health professionals			1.00	0.18*
Self-stigma				1.00

*p < 0.05; ***p < 0.001.

Intra-class correlation coefficients for test-retest reliability for each factor were as follows: informal networks [ICC 0.76 [IC 95% 0.67 -0.83]]; socio-institutional [ICC 0.62 [IC 95% 0.49-0.73]]; health professionals [ICC 0.62 [IC 95% 0.48-0.72]]; and self-stigma [ICC 0.74 [IC 95% 0.64-0.81]].

There were no significant differences in scoring with sex or age. Descriptively, service users in Argentina had the highest scores on almost all dimensions. As noted above, our study was not powered to explore differences in scoring between countries. However, for informal networks [Factor 1] this score was significantly higher than in Spain, Brazil and the UK; for socio-institutional stigma [Factor 2] this was higher than in the UK, Spain or Portugal; and for the factor health professionals stigma [Factor 3] the difference was significant in comparison to all the other countries [Table 4).

**Table 4:** **MARISTAN Stigma Scale**– **Factor scores by demography** [**Mean item score and** (**sd**)]

	**Informal networks**		**Socio-institutional**		**Health professionals**		**Self-stigma**	
	***M *** **( *SD *)**	***F***	***M *** **( *SD *)**	***F***	***M *** **( *SD *)**	***F***	***M *** **( *SD *)**	***F***
Total sample	2.9 (1.3)		3.7 (1.3)		2.5 (1.5)		3.7 (1.7)	
Gender								
Men	2.8 (1.3)	0.55	3.7 (1.3)	0.06	2.6 (1.5)	0.28	3.6 (1.6)	1.34
Women	3.0 (1.4)		3.6 (1.3)		2.5 (1.6)		3.9 (1,9)	
Age group								
18-29	2.4 (1.1)	2.50	3.5 (1.4)	0.73	2.2 (1.4)	1.93	3.5 (1.4)	1.43
30-39	2.8 (1.4)		3.6 (1.4)		2.5 (1.5)		3.4 (1.6)	
40-49	3.2 (1.3)		3.9 (1.3)		2.8 (1.6)		3.9 (1.8)	
50 and more	3.0 (1.3)		3.6 (1.2)		2.0 (1.3)		4.1 (1.8)	
Country								
Argentina	3.9 (1.2)	3.10*	4.7 (0.8)	3.36**	3.9 (1.1)	4.28**	4.1 (1.9)	2.08
Brazil	2.6 (1.2)		3.9 (1.5)		2.6 (1.7)		3.5 (1.8)	
Chile	3.2 (1.4)		4.0 (1.1)		2.7 (1.5)		4.4 (1.5)	
Portugal	2.9 (1.4)		3.6 (1.3)		2.3 (1.2)		3.2 (1.3)	
Spain	2.6 (1.1)		3.3 (1.3)		2.6 (1.3)		3.2 (1.7)	
UK	2.7 (1.3)		3.3 (1.4)		2.0 (1.5)		3.6 (1.8)	

*p < 0.05; **p < 0.01; ***p < 0.001.

## Discussion

We have developed a reliable instrument to measure the stigma of severe mental illness across six countries, and its cross-cultural, bottom-up development provides the validity needed to compare different dimensions of the stigma across cultures. It has moderate to high internal consistency [Cronbach’s Alpha 0.89] and moderate test-retest reliability [ICC 0.77]. There are four factors in the scale, namely ‘informal networks’, ‘socio-institutional’, ‘health professionals’ and ‘self-stigma’. However, because of highly variable correlations between them we recommend using the four separate subscale scores rather than a total score from the sum of all of them. Given that the ‘self-stigma’ subscale contains only four items, it could also be argued that an alternative scale (e.g. Ritsher et al.) [21] should be used if this were the main focus of a study. However, it is worth cautioning that test-retest reliability of this instrument was assessed in only 16 participants.

The questionnaire was applied by interviewers to make sure the items were understood and to help with any difficulties. Although this increased both time and costs associated with its application, it standardized data collection in diverse contexts, where the cultural background of the patients was very different. It now needs to be tested in a pure self-report format.

### Social networks

Patients’ social networks can be both a source of support and of stigma. Stigma complicates recovery and reduces self- esteem. When it arises in a person’s close relationships, it can have a severe knock-on effect on the take-up of opportunities for work, use of services, symptoms, hospitalization, trajectory of the illness, and quality of life. Although the family may be victim of stigma because one of its members has a mental illness [20], family members themselves can hold stigmatizing attitudes. The relationship between both processes is not yet clear; however recent research indicates that as people with schizophrenia become increasingly isolated, their family becomes the closest and most important support network. This may mean that patients are focused on this intimate relationship, even when it risks becoming a source of difficulties [33]. Few scales cover this issue in detail and it might be most relevant in societies with a lesser developed community mental health system.

### Stigma in the area of professional care

Unfortunately, formal care givers appear to be an important source of stigma. Two of the factors [socio-institutional and stigma from health professionals] touched on this source of stigma. Thus it may be important to include both the system and professionals when developing strategies to reduce stigma. A frequent complaint against health professionals concerns their attitudes, while perceptions of socio-institutional stigma concern processes of care and scarcity of resources. The latter requires political and public health solutions in each country. Stigma arising from professionals and institutions may be less likely to be experienced by patients with less than three years of illness or those who have never been institutionalised. One modification when applying the Maristán scale to this group is to use only the subscales ‘informal social networks’ and ‘self-stigma’ , both of which were strongly correlated with each other and both of which tapped important, albeit incomplete elements, of perceived stigma in this group of people.

The relation between stigma and the demand for mental health care does not appear to be direct. A number of authors have suggested that perceived stigma may not affect demand for mental health care in particular populations, but this may vary with the characteristics of the population affected [46]. Furthermore, people with schizophrenia are in long-term contact with specialized services and if they perceive stigma arising from such institutions and from health professionals, specific measures need to be taken to address it.

Both families and professionals were sources of stigma. Previous research is contradictory on this issue. There have been suggestions that close interaction with patients will reduce stigmatising or negative attitudes [47], however more recent research indicate that those with the closest contact may be the most discriminating of all [33]. Thus, we need closer study of just how the degree of intimacy leads to an increase or decrease in stigma.

### Types of stigma and scoring

Of the four factors we derived, three identify sources of stigma, while self-stigma, one of the most commonly found in other instruments to measure stigma [12,21], is the internalization of the public stigma; it is the experience of the self [17]. Our qualitative work however, showed that the other three areas of stigma were of much greater concern to patients and carers and thus this section of the scale is brief. The items in the self-stigma subscale capture internalized or subjective stigma and they reflect the prejudice which people with mental illness may turn against themselves [21], rather than explicit behaviours which give rise to discrimination. Although it includes a general item “people don’t respect me”, stigma perceived as arising from informal social networks included mainly family and other close persons and reflects the limited social networks which patients with psychotic mental illness can depend on. The fact that both factors [self-stigma and informal networks] are the most stable over time and have the greatest intra-factor correlation suggests they are closely related.

As opposed to other scales [14] there were no positive elements associated with suffering a severe mental illness. This may reflect the way in which the qualitative data were collected. In focus group discussions about stigma; patients and carers may be most concerned about discussing what is wrong in their lives rather than what is going well.

Users of services in Argentina, a country where psychiatric care is still largely concentrated in hospitals, had higher (worse) mean scores on the factors ‘informal networks’ , ‘socio-institutional’ and ‘health professionals’ than users in the United Kingdom, Spain, Portugal and Brazil, where community care appears to be better developed. This suggests that community based service provision is less stigmatising and professionals’ attitudes are less institutionalised and hierarchical, than in hospital based services.

### Limitations

There are a number of limitations to our study. First, the instrument has been tested in patients in contact with mental health services, a population that may have different perceptions of stigma than those who have no contact with services. Second, the questionnaire was validated in a relatively middle aged population that had been in contact with community services for a mean of over 12 years but had not had experience of long stay hospital care. We explain our reasons for that above. Third, the interviewers noted that a 5 point response scale might be simpler and thus the pattern of scoring may be revised in later testing of the instrument. Finally although we did not include any measure of criterion validity, we omitted this for two reasons: 1. No stigma scale exists that has universal application across these six cultures and 2. We believe that its multicultural, bottom-up development provides the validity needed.

### Future work

We have presented the development and standardisation of the MARISTAN Stigma Scale. Further evidence on its structure, in particular confirmatory factor analyses, is needed, as well as its application in treatment services for schizophrenia and related psychoses around the world.

## Conclusion

The MARISTAN stigma scale is a reliable measure of the stigma of schizophrenia and related psychoses across several cultures. A confirmatory factor analysis is needed to assess the stability of its factor structure.

## Competing interests

The authors declare that they have no competing interests.

## Authors’ contributions

FT-G, MK, SS, MX, EG, JMC, AR-G, BV: Conceived of and designed the study. MK, HK, RG, AR-G, MX, PG, CA, DAB: Coordinated the collection of data in each country. SS and MK: wrote the article and PG worked on the draft. FT-G, EG, BV, JMC, AR-G, MX, PG, CA, DAB: Gave final approval of the version to be published. RM: undertook the statistical analysis and helped to interpret the findings. All authors read and approved the final manuscript.

## Pre-publication history

The pre-publication history for this paper can be accessed here:

http://www.biomedcentral.com/1471-244X/14/182/prepub
